# HPV integration profiling using nanopore sequencing and association with cervical precancerous lesion

**DOI:** 10.3389/fmicb.2025.1522550

**Published:** 2025-03-05

**Authors:** Ying Hou, Shoufeng Ni, Xin Liu, Xingyu Liu, Nan Wang, Fuqiang Xu, Jianyong Gao, Yanmei Li, Yuxiang Zhou, Huadong Tang, Meina Bian, Xiulan Li, Lili Zhang, Weiwei Wang, Qing Liu

**Affiliations:** ^1^Department of Gynecology, Beijing Youan Hospital, Capital Medical University, Beijing, China; ^2^Geneis Beijing Co., Ltd., Beijing, China; ^3^CapitalBio Technology Corporation, Beijing, China

**Keywords:** human papillomavirus, cervical lesions, cervical cancer, nanopore sequencing, virus integration

## Abstract

**Objectives:**

HPV infection and HPV DNA integration can lead to cervical cancer, but the relationship with lesion severity is unclear. This study aimed to investigate the correlation between HPV integration profile and cervical lesion extent.

**Materials and methods:**

Twenty patients representing cervicitis, CIN I, CIN II, and CIN III underwent nanopore sequencing for HPV genotype and integration site analysis. HPV integration profiles were correlated with lesion severity. Gene Ontology (GO) and KEGG analysis were used to identify stage-specific genes and pathways.

**Results:**

HPV integration rates were 60, 60, 100, and 100% for cervicitis, CIN I, CIN II, and CIN III, respectively, with varying numbers of integrated genes. Each group had specific stage-related genes, with 83 shared genes linked to neuron development and cell–cell processes. CIN II and CIN III displayed more cancer-related pathway enrichment than earlier stages.

**Conclusion:**

A positive correlation exists between HPV integration frequency and cervical lesion stage. Late-stage lesions showed heightened enrichment in cancer-related pathways through specific HPV-integrated genes.

## Introduction

Cervical cancer is a common malignant tumor of the female reproductive system. More than 520,000 new cases and 260,000 deaths due to cervical cancer were reported worldwide in 2020 (Siegel et al., 2020). The incidence of cervical cancer in Chinese women accounted for about 30% of the world’s new cases (Serkies and Jassem, 2018). In China, there is a steady rise in cervical cancer cases, particularly among younger individuals (Afaya et al., 2022). In 2018, there were more than 100,000 new cases of cervical cancer and over 40,000 deaths among Chinese women. Fortunately, deaths from cervical cancer are largely preventable through screening, early diagnosis, and timely treatment (Pimple and Mishra, 2019). Recently, a three-step screening procedure was adopted for cervical cancer screening in China (Tsikouras et al., 2016). The screening involves detecting human papillomavirus (HPV), thin-layer cytology (TCT), and colposcopy histopathological biopsy. Nonetheless, the current screening methods have a low positive predictive value for cervical precancerous lesions, and the specificity of high-risk HPV (HR-HPV) results is low (Women Health Care Branch of Chinese Preventive Medicine Association, 2017). Therefore, new screening methods are needed to avoid misdiagnosis, over-diagnosis, and over-treatment.

HPV infection, especially persistent HR-HPV infection, is closely related to the occurrence and development of cervical lesions (Crosbie et al., 2013; Hu and Ma, 2018). However, the progression from HPV infection to cervical cancer remains poorly understood, which typically takes several years or decades (Hu and Ma, 2018). HPV can integrate into the host genome, causing cervical lesions by silencing tumor suppressors or activating tumor promoters (Olusola et al., 2019). HPV integration is a key factor in the occurrence and development of cervical cancer (Huang et al., 2017; Balasubramaniam et al., 2019). It suggests that it has the potential to predict the development of cervical cancer, which could facilitate clinical intervention and follow-up.

There are several limitations to the current HPV detection methods. Pap smears have a high false-negative rate, and thin-layer liquid-based cytology has suboptimal sensitivity and specificity (Jin et al., 2024). The low specificity of screening tests can lead to women being either under-or overtreated (Abreu et al., 2012). The available molecular biological techniques are complex and time-consuming, and some techniques (e.g., southern blot, northern blot, and reverse dot blot) require relatively large amounts of purified DNA (Jin et al., 2024). *In situ* hybridization is used for tissues. Polymerase chain reaction is limited by the intrinsic instability of RNA. Microarrays have promising features, but their cost limits their wide-scale use (Jin et al., 2024). Hybrid capture (HC) is widely used in the screening setting, but HCI has low specificity, and HCIII cannot subtype HPV (He et al., 2020). Those techniques detect HPV and do not detect host integration.

Various techniques are currently used to detect HPV integration, including amplification of papillomavirus oncogene transcripts (APOT), detection of integrated papillomavirus sequences (DIPS-PCR), and next-generation sequencing (NGS) (Luft et al., 2001; Olthof et al., 2015; Tuna and Amos, 2017). However, APOT may have limited sensitivity in detecting low levels of integrated HPV, DIPS-PCR is consuming and laborious, and NGS may generate false-positive results due to sequencing errors, requiring rigorous data filtering and validation (Gradíssimo and Burk, 2017; Yang et al., 2020). The profiling of the large-range HPV integration sites is still far from completed using previous assays. Nanopore sequencing is a single-molecule third-generation sequencing technology with advantages such as long sequencing read length, real-time sequencing and analysis, and easy portability (Wang et al., 2021). Nanopore sequencing has also been used to detect long-segment nucleic acid structural variation and virus insertion sites (Croville et al., 2018; Bull et al., 2020). This method has been confirmed to possess superior sensitivity, accuracy, and read length. Combining this method with capture technology could enhance the sensitivity of HPV integration site detection, enabling the acquisition of more comprehensive and complete information regarding HPV integration sites.

In this study, we used a probe-capture-based method to enrich HPV DNA fragments and sequenced by third-generation nanopore sequencer to simultaneously identify HPV genotype and integration sites in cervical lesion samples at different stages, allowing us to evaluate the correlation between HPV integration profiling and the extent of cervical lesions. The results of this study could provide a possibility of using nanopore-based technology as an assay for early screening and diagnosis of cervical lesions.

## Materials and methods

### Specimen source

A total of 20 samples were selected from the gynecology department of Beijing Youan Hospital affiliated to Capital Medical University, between January 2020 and December 2021, including five specimens each of cervicitis (simple HPV infection), cervical intraepithelial neoplasia (CIN) I, CIN II, and CIN III, respectively. The classification criteria for cervicitis, CIN I, CIN II, and CIN III are listed in Supplementary Table S1. Informed consent was obtained from the patients for all samples included in this study. The study protocol was reviewed and approved by the Medical Ethics Committee of Beijing Youan Hospital affiliated to Capital Medical University (Ethics Number: LL-2019-91-K).

### DNA extraction and fragmentation

Exfoliated cervical epithelial cell samples were collected by a set of disposable sampling brushes of the cervix and uterine cavity (Meijiajia, Xi’an, China). Genomic DNA (gDNA) was extracted using the TIANamp Micro DNA Kit (Tiangen, Beijing, China) according to the manufacturer’s protocol. Double-stranded (ds) DNA was quantified using the Qubit dsDNA HS Assay Kit (Thermo Fisher Scientific, Inc., Waltham, MA, United States). One hundred and fifty nano grams of gDNA was sheared to 1–3 kb using the Covaris M220 (Covaris, LLC, Woburn, MA, United States). Fragmented gDNA was examined on an Agilent BioAnalyser 2100 (Agilent Technologies Inc., Palo Alto, CA, United States) to ensure the gDNA material has the expected size distribution.

### Nanopore library construction

The nanopore library was constructed using a nanopore PCR Barcoding Kit (SQK-PBK004, Oxford Nanopore Technologies) and its recommended third-party reagents, NEBNext Ultra II End repair/dA-tailing Module (E7546) for end-prep, LongAmp Hot Start Taq 2X Master Mix (NEB, M0533S) for PCR adapters ligation and amplification. The methods were carried out according to a modified protocol. Fragmented gDNA was added to the end-prep reaction at 20°C for 30 min, then 65°C for 30 min using a thermal cycler. The nanopore adapter ligation was performed at 25°C for 15 min. The number of amplification cycles was adjusted to generate more than 500 ng of library for further hybridization.

### Hybridization, capture, and sequencing

Nanopore library (500 ng) was hybridized with HPV probes (BOKE bioscience, Wuxi, China) at 65°C for 16 h with other steps following the manufacturer’s instructions. The captured library was amplified with the same barcode primers for library construction for 30 cycles to generate a sequencing library. The library was quantified using a Qubit dsDNA HS Assay Kit (Thermo Fisher Scientific, Inc., Waltham, MA, United States), and the final size of the library was determined using an Agilent BioAnalyser 2100 (Agilent Technologies Inc., Palo Alto, CA, United States).

The nanopore library was sequenced using the PromethION platform (Oxford Nanopore Technologies; Oxford, United Kingdom) according to the manufacturer’s protocol, with the *Q*-score value set at ≥7. The quality control analysis for the nanopore sequencing is shown in Supplementary Table S2.

### Bioinformatic analysis

We used the Guppy software (Oxford Nanopore Technologies) for base calling, then used NanoPack (De Coster et al., 2018) to filter the sequencing reads with parameters *Q*-value ≥7 and sequence length more than 500 bp. The filtered clean reads were aligned against the human reference genome (hg 19) and HPV genome databases using an in-house data analysis pipeline to obtain the information on breakpoint and integration sites.

Functional enrichment analysis was performed on the genes associated with insertion sites using the clusterProfiler package in R.^1^ Gene Ontology (GO) and Kyoto Encyclopedia of Genes and Genomes (KEGG) pathways were assessed. A pathway or GO term was considered significantly enriched if it met the criteria of a corrected *p*-value (Benjamini–Hochberg adjusted) less than 0.05 and a *Q*-value (false discovery rate) less than 0.05.

## Results

### HPV integration ratio and frequencies

The basic information of the patients who provided the clinical samples is listed in Supplementary Table S3. There were five samples in each group: Cervicitis, CINI, CINII, and CINIII. The groups were compared to each other, but there was no control group without HPV infection.

**Figure 1:**
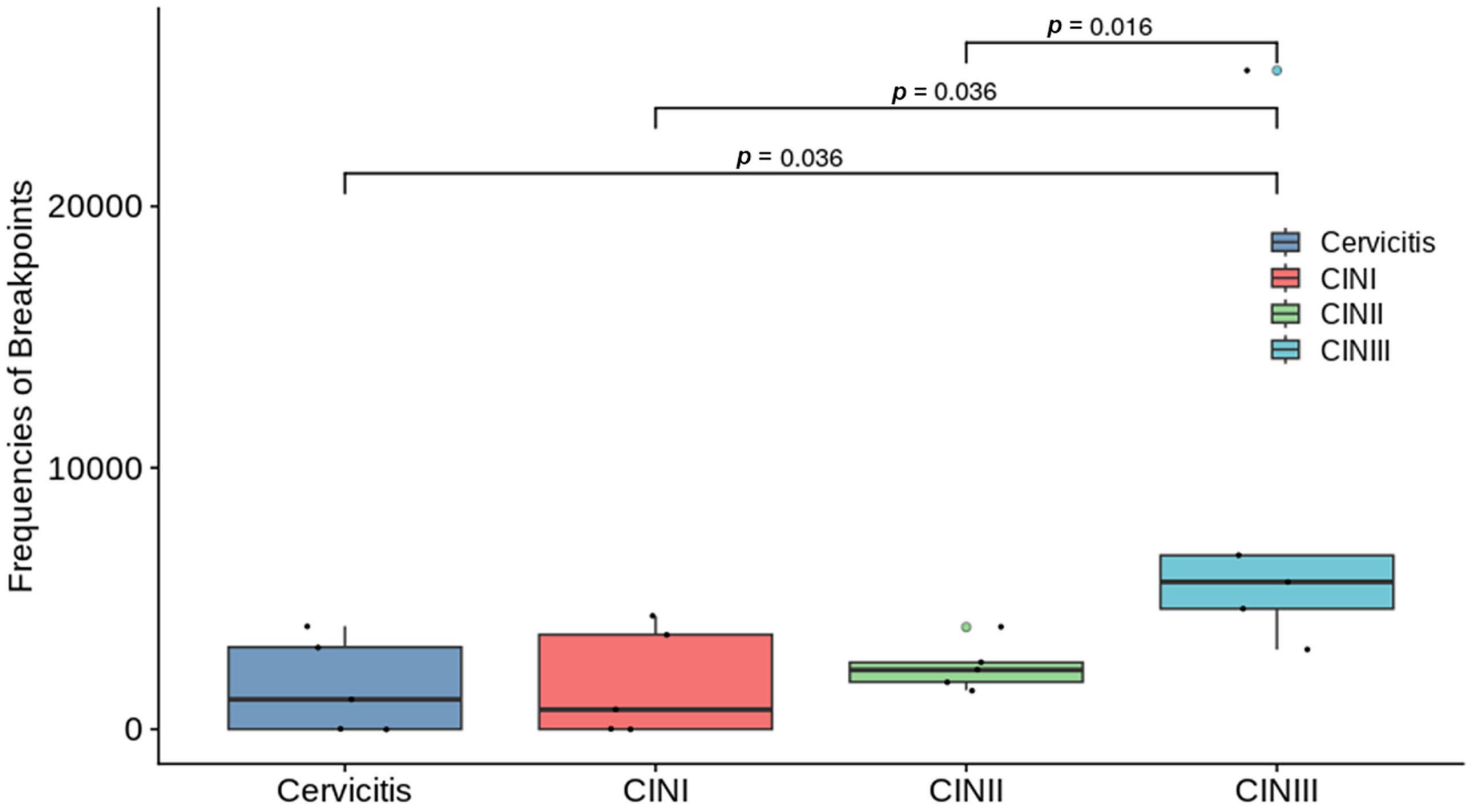
Graph comparing the breakpoint frequencies between the groups with different stages of cervical infection: cervicitis, cervical intraepithelial neoplasia (CIN) I, CIN II, and CIN III groups. Where differences were statistically different, they are indicated on the graph with a line and the corresponding *p*-value. No statistical differences were found among the other groups.

The HPV-positive reads with the target type (Table 1) were identified in all the samples. The rates of positive samples with HPV integration sites in the Cervicitis, CINI, CINII, and CINIII groups were 60, 60, 100, and 100%, respectively. The average frequencies of breakpoints in the four groups were 1645.21, 1739.92, 2403.79, and 9026.15, respectively (Table 1). Statistically significant differences in breakpoint frequency were observed between CINIII and cervicitis, CINI, and CINII, respectively (*p* = 0.036, *p* = 0.036, *p* = 0.016), while no statistical differences were found among cervicitis, CINI and CINII groups (Figure 1). This result suggested that there was an increasing trend of HPV integration along the course of the disease.

**Table 1:** Detected integrations per sample.

Cervical lesion stage	Sample #	HPV subtype	Number of HPV reads	Number of HPV integration sites	Frequency of breakpoints^a^
Cervicitis	A03	HPV18	1,911	6	3139.72
	A13	HPV16	44	0	0
	A17	HPV16	5	0	0
	A14	HPV56	1,018,558	1,160	1138.86
	A25	HPV18	61,812	244	3947.45
	Average				1645.21
	HPV integration rate			60%	
CINI	A01	HPV52	7	0	0
	A20	HPV58	30,254	131	4330.01
	A21	HPV39	13	0	0
		HPV58	48	0	
	A02	HPV56	9,341	7	749.38
	A23	HPV56	1,466,774	5,310	3620.19
	Average				1739.92
	HPV integration rate			60%	
CINII	A05	HPV52	837,581	2,139	2553.78
	A07	HPV56	243,191	551	2265.71
	A12	HPV31	1,181,054	2,134	1806.86
	A16	HPV33	1,415,230	2,102	1485.27
	A26	HPV18	54,257	212	3907.33
	Average				2403.79
	HPV integration rate			100%	
CINIII	A10	HPV16	754	19	25,198.94
	A15	HPV58	453,599	1,380	3042.33
	A24	HPV16	1,360	7	5633.8
		HPV68	60	1	
	A09	HPV59	745,756	3,135	4606.06
	A27	HPV16	43,010	286	6649.62
	Average				9026.15
	HPV integration rate			100%	

HPV, human papillomavirus; CIN, cervical intraepithelial neoplasia.

a Frequency of breakpoints = number of breakpoints/number of reads × 10^6^ (the number of breakpoints per megareads).

### Distribution of integration sites in the human genome

**Figure 2:**
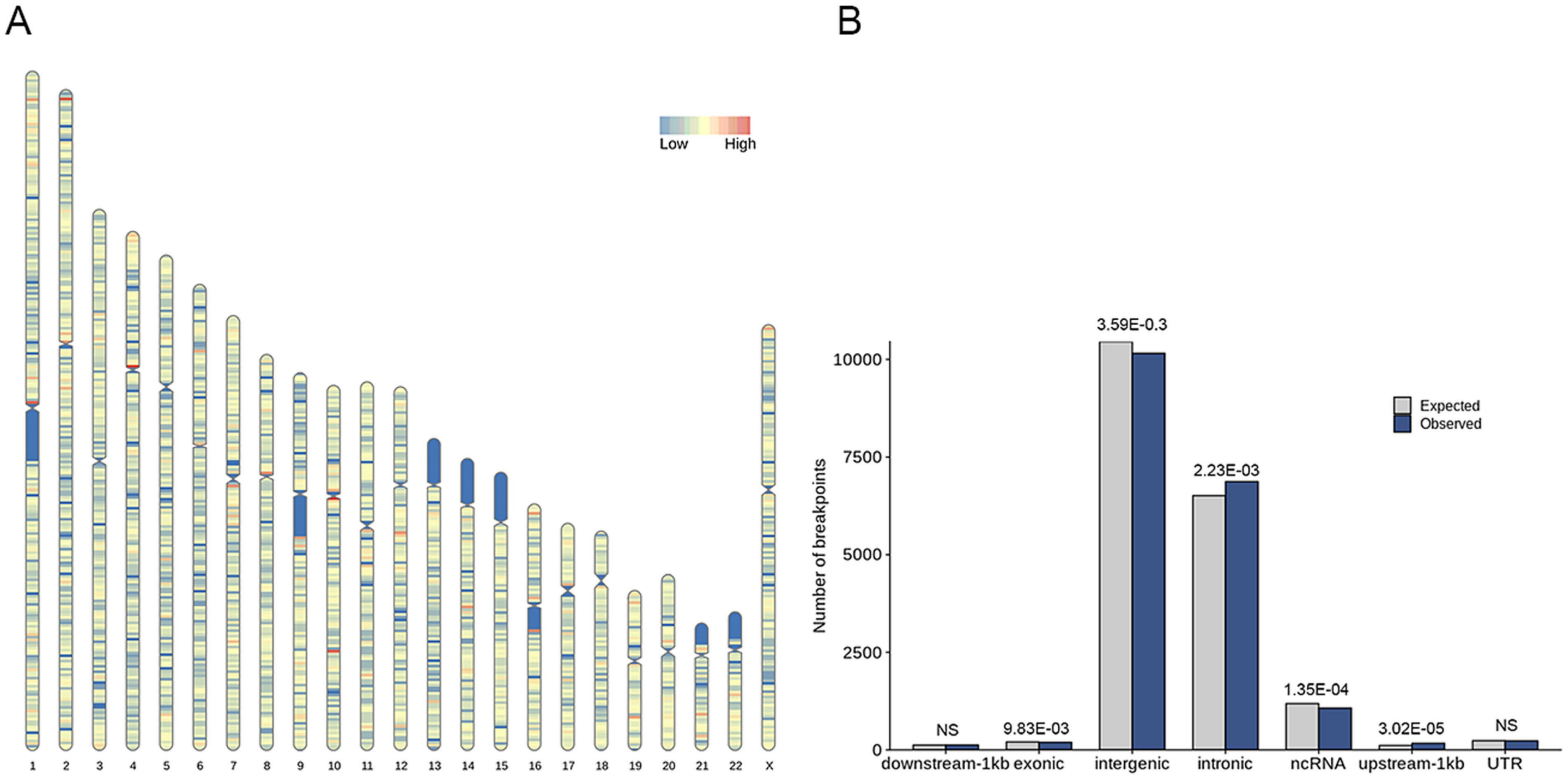
Distribution of HPV integration sites within the human genome. **(A)** Graphic representation of the distribution of HPV integration sites on chromosomes. The integration rate is color-coded, with blue representing a low degree of integration and red representing a high degree of integration (more than 10 integrations within 1 Mb region). **(B)** Distribution of HPV integration sites in annotated regions of the human genome. The expected (assuming uniform, random distribution, grey) and the observed (actual numbers, blue) number of HPV integration sites are shown for each location.

A total of 18,824 HPV integration sites were identified and annotated on the human genome. It showed 259 hotspots (more than 10 integrations within 1 Mb region) in the human genome (Figure 2A), indicating that the integration of HPV might be region or sequence-specific. The HPV integration sites were further annotated and categorized as intergenic, intronic, ncRNA, UTR, exonic upstream-1 kb, and downstream-1 kb. The proportions of integration at these locations were 53.99, 36.51, 5.69, 1.23, 1.03, 0.90 and 0.66%, respectively (Supplementary Table S4). Analysis of these integrations suggested that the number of observed integrations in the intronic (*p* = 2.23 × 10^−3^, One sample *t*-test) and upstream (within 1 kb) locations of genes (*p* = 3.02 × 10^−5^, One sample *t*-test) was significantly higher than expected (Figure 2B).

### The HPV-integrated genes of the different groups

**Figure 3:**
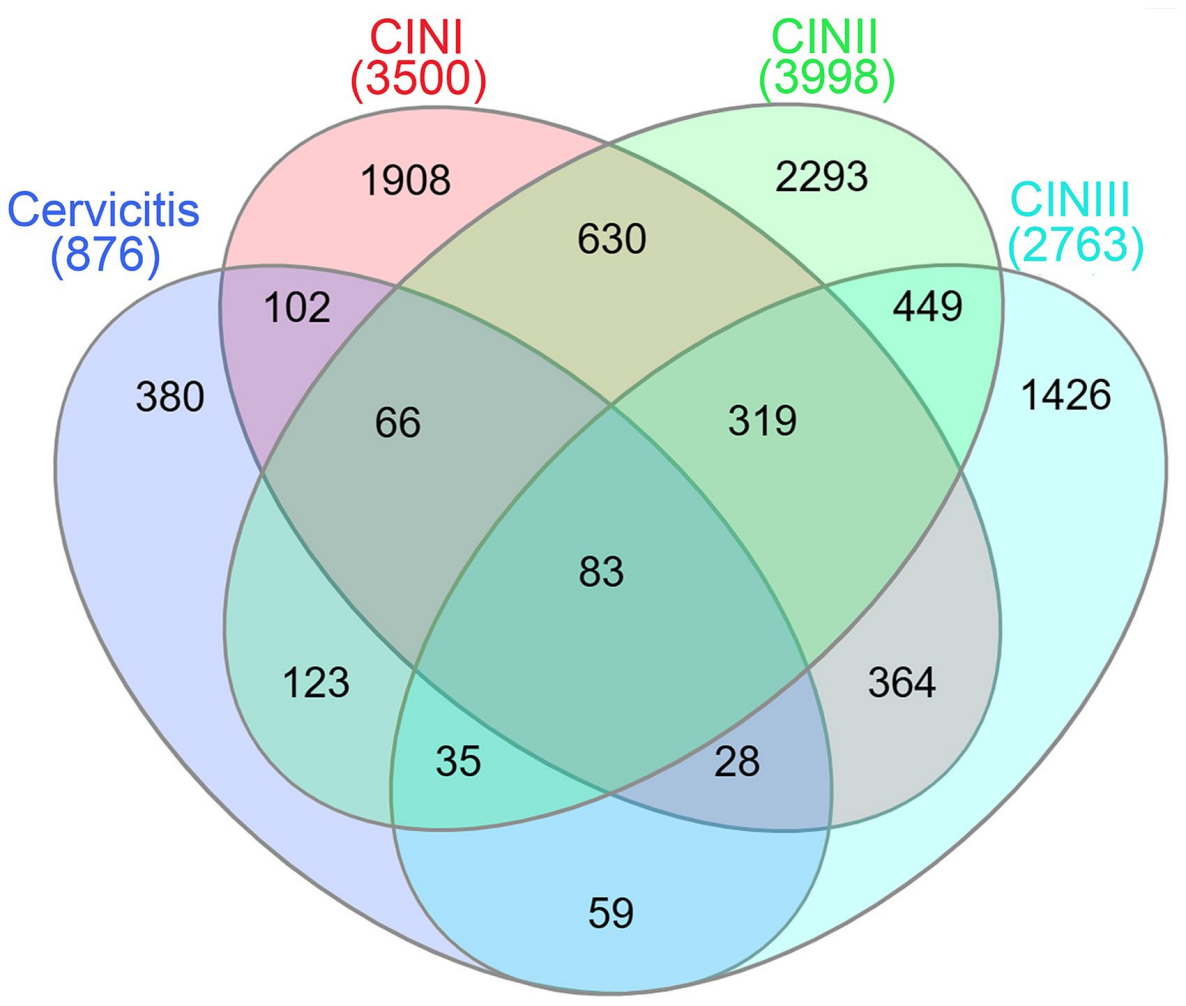
Venn diagram showing the over-lapping HPV-integrated genes and potential stage-specific markers obtained from analysis of the Cervicitis, CIN I, CIN II, and CIN III groups.

The HPV-integrated genes detected by third-generation nanopore analysis in the Cervicitis, CIN I, CIN II, and CIN III groups were 876, 3,500, 3,998, and 2,763, respectively. A Venn diagram was generated to identify genes overlapping the different groups. The analysis revealed that 83 genes were shared by all four groups. There were 380, 1,908, 2,293, and 1,426 unique HPV-integrated genes in the Cervicitis, CIN I, CIN II, and CIN III groups, respectively (Figure 3).

**Figure 4:**
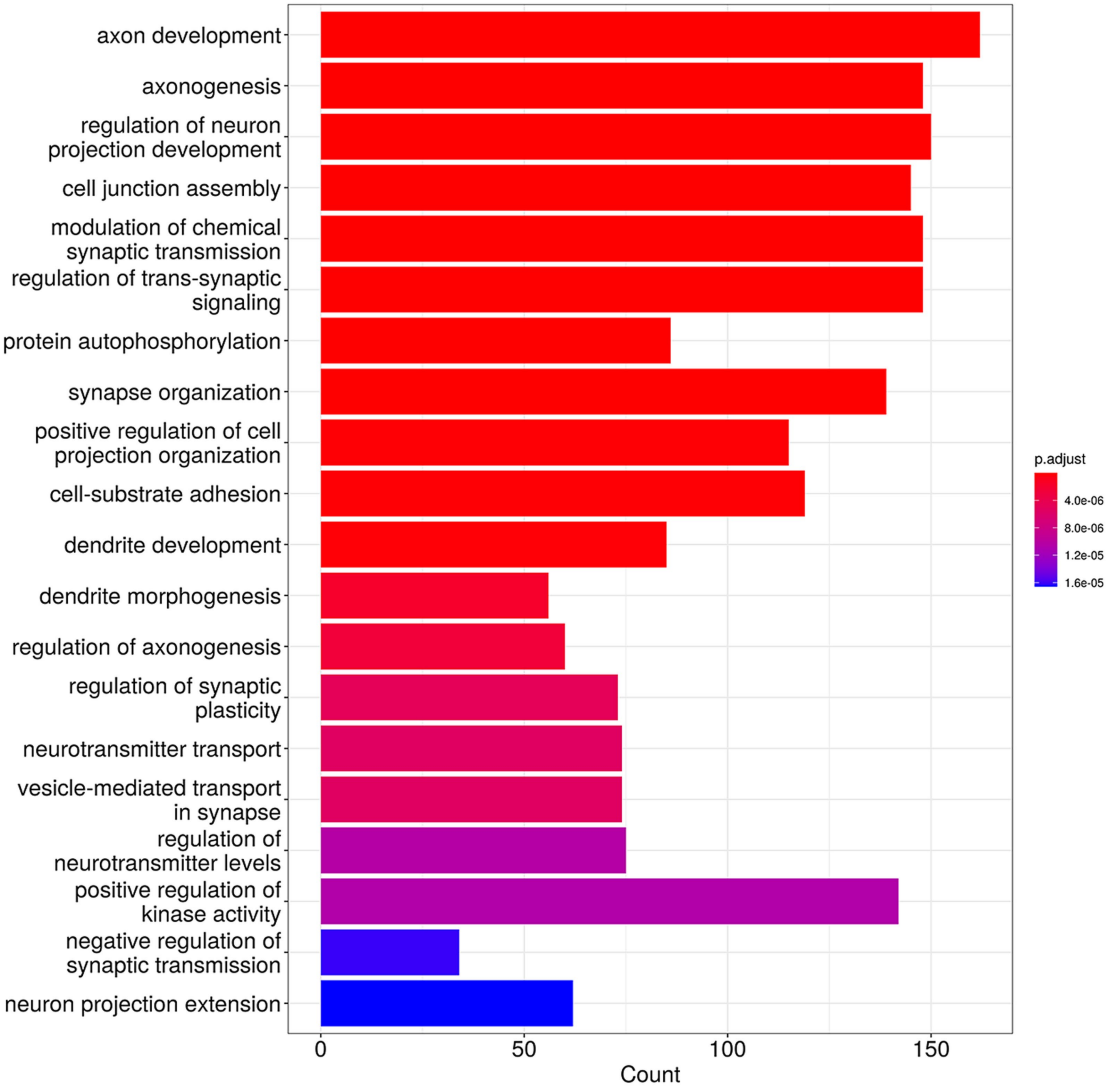
Gene Ontology (GO) analysis of the 83 HPV-integrated genes shared among four groups with different cervical lesion stages showing the top 20 enriched functional categories.

The results of GO analysis in the biological process category suggested that the 83 genes common to all four groups were mainly enriched in the biological processes related to the regulation of neuron development and cell–cell adhesion/interaction (Figure 4). The unique genes for each group were also analyzed, and the results are presented in Supplementary Table S5. This analysis suggested that cervicitis stage-specific genes were primarily enriched in apoptotic-related processes and chronic inflammatory response; stage-specific CIN I genes were mainly enriched in processes related to cell cycle regulation and metabolic pathways; stage-specific CIN II genes were mainly enriched in DNA and chromosome-related repair and organization functions; stage-specific CIN III genes were mainly enriched in cancer pathways, including Wnt signaling, cell–cell adhesion, and others.

**Figure 5:**
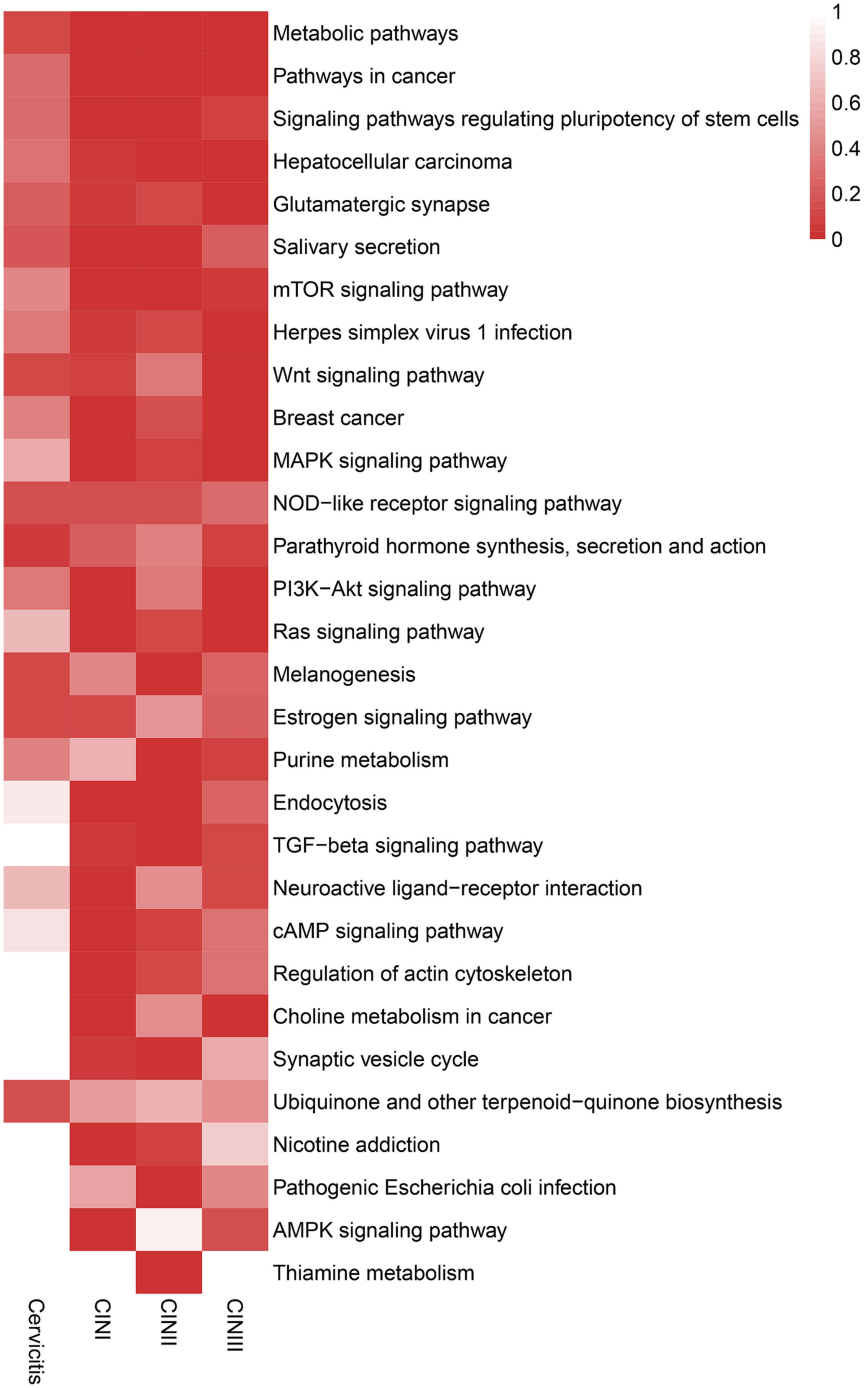
Heatmap showing the enriched KEGG pathways of the stage-specific integrated genes of the cervicitis, CIN I, CIN II, and CIN III groups. The stage-specific integrated genes showed more concentration in cancer-related signaling pathways in later stages of CIN II and CIN III than in earlier stages. A darker red color indicates greater enrichment.

According to the KEGG pathway analysis, the enriched pathways of the stage-specific integrated genes in the four stages were consistent with the GO analysis. In the later stages, CIN II and CIN III, there was more enrichment in cancer-related signaling pathways, including transforming growth factor (TGF)-beta, mammalian target of rapamycin (mTOR), mitogen-activated protein kinase (MAPK), and RAS signaling as shown in a heatmap (Figure 5).

## Discussion

HPV-positivity strongly correlates with poor prognosis of cervical cancer (Lei et al., 2022). In recent years, HPV detection has been deemed important to prevent and control cervical cancer. Currently, HPV DNA is found in nearly all cervical cancer lesions (Bhatla and Singhal, 2020). It has been reported that the integration of high-risk HPV DNA into the human genome significantly contributes to cervical carcinogenesis (Oyervides-Muñoz et al., 2018). Nevertheless, the relationship between HPV integration and cervical cancer remains unclear. Nanopore sequencing is a novel and valuable approach that does not require amplification and fluorescence labeling and has striking advantages in reliability and resolution compared to NGS (Laszlo et al., 2013). More significantly, nanopore sequencing enables the simultaneous detection of HPV and bacteria (Quan et al., 2019) and the identification of more HPV types (Chan et al., 2020). In this study, we conducted a comparative analysis of HPV integration profiles from precancerous cervical lesions of 20 patients using nanopore long-read sequencing. Our findings reveal a positive correlation between the stage of cervical lesion and the prevalence of HPV integrations, along with the average frequency of integration sites in these samples. Therefore, it may indicate that HPV integration plays a crucial role in promoting the progression of cervical lesions.

The development of cervical cancer, including precancerous lesions, CIN grades I–III, and early infiltrating cancer, requires a relatively long time (Ren et al., 2021). Most HPV infections are transient and can be spontaneously cleared by the immune system within 1–2 years, while a few persist for many years or even decades (Stanley, 2010). Additionally, HPV detection was able to identify abnormalities in cervicitis and CIN grades I–III during follow-up, but not TCT, suggesting the superior accuracy of HPV detection compared to TCT detection.

By analyzing specific HPV-integrated genes, 83 genes were common to the Cervicitis, CIN I, CIN II, and CIN III groups. The GO and KEGG analysis indicated these genes may participate in basic physiological activities and cell–cell adhesion/interaction. They may contain hotspot regions or sequence motifs that attract viruses to integrate. We also identify 380, 1908, 2,293, and 1,426 unique HPV-integrated genes in the Cervicitis, CIN I, CIN II, and CIN III groups, respectively. These genes may contain potential features for identifying stage-specific biomarkers in different stages of cervical lesions. The results of GO and KEGG suggested that the unique genes in the cervicitis group were concentrated in cell damage and repair, while the genes in the CIN groups gradually focused more on regulating the cell cycle, signaling pathway, and inflammatory factors. In addition, these genes were involved in various physiological activities, including cell migration, adhesion, division, proliferation, differentiation, and apoptosis, closely related to tumorigenesis. The higher the cervical lesion grade, the more likely the unique genes would be enriched in carcinogenesis-related functions.

Although HPV integration has been documented as an important factor in the progression of cervical lesions (Balasubramaniam et al., 2019; Olusola et al., 2019), these studies have relied mainly on short-read sequencing methods and may not accurately characterize the global characteristics of integration sites. In particular, low-frequency integration events and changes in the function of upstream and downstream genes are often overlooked. In the present study, nanopore sequencing technology was used to capture integration events more comprehensively, providing a new perspective for further revealing the integration mechanism. Furthermore this study explored the role of HPV integration properties in different stages of cervical lesions, which requires technologies that can detect a wide range of integration sites. Nanopore sequencing provides long read lengths, but it also reduces bias in traditional sequencing steps through real-time analysis (Wang et al., 2021). This technology enables this study to reveal for the first time the systematic distribution of HPV integration sites and their potential role in cancer progression. In addition, nanopore sequencing has shown significant advantages in HPV integration detection due to its long read length and real-time analysis capabilities (Bull et al., 2020; Wang et al., 2021). Compared with traditional methods such as APOT and DIPS-PCR, nanopore sequencing is more suitable for detecting complex viral integration sites and structural variants. Nevertheless, its high error rate may affect the accuracy of the results, which needs to be optimized by rigorous quality filtering and subsequent correction tools such as Nanopolish (Yang et al., 2020). In addition, traditional methods such as DIPS-PCR have high specificity but are complex and have a limited detection range, making it difficult to analyze genome-wide integration sites. Finally, the findings of this study are consistent with previously reported HPV integration patterns in cancer (Laszlo et al., 2013; Oyervides-Muñoz et al., 2018). However, the present study contrasts with the conclusion of a previous study that HPV integration was not found to significantly affect lesion progression (Yang et al., 2020). The differences possibly stem from the different detection methods. For example, traditional short-read sequencing techniques may struggle to capture a complete picture of the viral insertion sites, especially for low-abundance integration events. The advantage of nanopore sequencing is that it can more comprehensively detect long variants, thereby revealing a broader range of viral integration properties.

The present study has some strengths. This study explored the relationship between HPV integration profiles and the extent of cervical lesions, providing a promising diagnostic method for assessing cervical cancer progression using nanopore sequencing. GO and KEGG pathway analyses were performed to identify stage-specific genes and pathways at different stages, providing new insights into the pathogenesis of cervical cancer. Finally, third-generation nanopore sequencing technology was used, capable of detecting HPV genotypes and integration sites simultaneously with high sensitivity and accuracy.

Nonetheless, this study still has some limitations. Firstly, although the prevalence and frequency of HPV integrations were positively correlated with disease progression, the sample size in this study was small, and there was no control HPV-negative group. Nevertheless, the concept of nanopore sequencing for detecting integrated HPV genome had to be validated in patient specimens before conducting a large-scale, multicenter validation study, which is currently recruiting. That multicenter study will also allow the investigation of HPV subtypes and their integrative properties, which was not possible in the present study due to the small sample size. Secondly, the follow-up period for the patient in this study was short and ongoing. A longer follow-up duration is necessary to assess the progression, recurrence, and survival rates and the correlation analysis with HPV integrations. A prospective longitudinal study is necessary to determine the role of HPV integration in the pathogenesis of cervical cancer and establish causal relationships. Thirdly, with the stage-specific integrated genes and pathways identified in this study, additional extensive experiments are needed to investigate the molecular mechanisms involved in cervical cancer progression. Fourthly, the Guppy software was used for initial base recognition, and NanoPack was used to filter the low-quality reads, but no other corrective steps have been performed, which could affect the quality of the reads. Additional quality control methods and their impact on the results will be investigated in future studies. For now, the small sample size also prevents the analysis of how errors in sequencing can influence the classification of HPV integration.

In summary, this study provides a promising diagnostic assay using nanopore sequencing to evaluate cervical cancer progression for early screening. By identifying the integration profiles from different precancerous stages, we further identify potential stage-specific genes and pathways related to the progression of cervical cancers. However, further validation is still needed for the molecular mechanisms of specific HPV-integrated genes that triggered the progression of cervical lesions at different stages (Kurman et al, 2014). Future studies will include a larger sample size from multiple centers to validate the findings. In addition, the functional role of HPV-integrated genes and their specific mechanisms in the process of carcinogenesis need to be verified experimentally to reveal potential therapeutic targets. The hybrid strategy combining multiple sequencing platforms can also further improve the accuracy of integrated site detection, providing a stronger foundation for early diagnosis and personalized treatment.

## Data Availability

The datasets presented in this study can be found in online repositories. The names of the repository/repositories and accession number(s) can be found in the article/Supplementary material.

## Footnotes

^1^ www.r-project.org
