# Supplementary material for: HPV integration profiling using nanopore sequencing and association with cervical precancerous lesion
Source: Front Microbiol. 2025 Mar 5;16:1522550. doi: 10.3389/fmicb.2025.1522550 (PMC11920162; doi:10.3389/fmicb.2025.1522550)
Supplement: Supplementary file 1 [file Table_1.docx]

Table S1: Histological and pathological diagnosis results

| **Pathological Diagnosis Results** | **Cervicitis** | **CIN I** | **CIN II** | **CIN III** |
| --- | --- | --- | --- | --- |
| Nucleus | - | Enlarged | Pathological Mitosis | More pathological mitoses, loss of polarity |
| Cell morphology | - | Regular | Irregular | Heterogeneity and pleomorphism |
| Number of heteromorphic cells | - | ≦1/3 epithelial layer | ≦2/3 epithelial layer | >2/3 epithelial layer |

Table S2: Nanopore sequencing quality control results

| **ID** | **Total Bases (G)** | **Total Reads** | **Pass Bases** | **Pass Reads** | **Clean Bases** | **Clean Reads** | **Read mean length** | **Read length N50** | **Read mean quality** | **Pass Ratio (pass/total)** | **Clean Ratio (clean/pass)** |
| --- | --- | --- | --- | --- | --- | --- | --- | --- | --- | --- | --- |
| A03 | 1.18 | 1269727.0 | 1.1 | 1195418.0 | 1.05 | 1063323.0 | 986.7 | 1047.0 | 10.6 | 93.22% | 95.45% |
| A13 | 1.00 | 998690.0 | 0.94 | 945946.0 | 0.91 | 883145.0 | 1032.2 | 1090.0 | 10.6 | 94.00% | 96.81% |
| A17 | 1.10 | 909626.0 | 1.04 | 860854.0 | 1.02 | 813552.0 | 1253.6 | 1381.0 | 10.6 | 94.55% | 98.08% |
| A14 | 5.17 | 4938288.0 | 4.88 | 4640847.0 | 4.80 | 4441519.0 | 1079.7 | 1153.0 | 10.9 | 94.39% | 98.36% |
| A25 | 1.51 | 1514438.0 | 1.4 | 1420049.0 | 1.34 | 1263007.0 | 1062.3 | 1149.0 | 10.6 | 92.72% | 95.71% |
| A01 | 1.34 | 1478401.0 | 1.25 | 1392721.0 | 1.2 | 1249834.0 | 956.4 | 1000.0 | 10.6 | 93.28% | 96.00% |
| A20 | 1.52 | 1573864.0 | 1.42 | 1488095.0 | 1.36 | 1318946.0 | 1028.1 | 1102.0 | 10.6 | 93.42% | 95.77% |
| A21 | 1.23 | 966045.0 | 1.16 | 917044.0 | 1.15 | 876743.0 | 1307.8 | 1433.0 | 10.7 | 94.31% | 99.14% |
| A02 | 4.86 | 3498772.0 | 4.65 | 3332554.0 | 4.61 | 3247783.0 | 1420.3 | 1608.0 | 11.0 | 95.68% | 99.14% |
| A23 | 2.68 | 2583405.0 | 2.45 | 2384200.0 | 2.36 | 2129560.0 | 1106.9 | 1202.0 | 10.4 | 91.42% | 96.33% |
| A05 | 4.38 | 2785866.0 | 4.14 | 2629319.0 | 4.11 | 2572077.0 | 1599.1 | 1760.0 | 10.9 | 94.52% | 99.28% |
| A07 | 5.96 | 5922917.0 | 5.68 | 5616891.0 | 5.51 | 5200358.0 | 1058.9 | 1143.0 | 10.8 | 95.30% | 97.01% |
| A12 | 3.69 | 3019685.0 | 3.5 | 2856065.0 | 3.44 | 2715060.0 | 1266.1 | 1395.0 | 10.8 | 94.85% | 98.29% |
| A16 | 4.40 | 3625913.0 | 4.11 | 3376077.0 | 4.07 | 3270628.0 | 1244.3 | 1339.0 | 10.9 | 93.41% | 99.03% |
| A26 | 4.84 | 4916917.0 | 4.59 | 4643733.0 | 4.48 | 4365441.0 | 1025.6 | 1080.0 | 10.8 | 94.83% | 97.60% |
| A10 | 0.95 | 939229.0 | 0.89 | 888504.0 | 0.87 | 821281.0 | 1057.3 | 1126.0 | 10.6 | 93.68% | 97.75% |
| A15 | 1.14 | 1365875.0 | 1.06 | 1276382.0 | 0.98 | 1070732.0 | 913.5 | 952.0 | 10.4 | 92.98% | 92.45% |
| A24 | 1.23 | 1140577.0 | 1.15 | 1077637.0 | 1.12 | 1003909.0 | 1115.5 | 1205.0 | 10.6 | 93.50% | 97.39% |
| A09 | 1.28 | 1403603.0 | 1.19 | 1320424.0 | 1.14 | 1173402.0 | 969.6 | 1022.0 | 10.5 | 92.97% | 95.80% |
| A27 | 4.86 | 4215751.0 | 4.64 | 4011684.0 | 4.59 | 3888952.0 | 1180.9 | 1283.0 | 10.9 | 95.47% | 98.92% |

Table S3: Patient enrollment information

| **Lesion stage** | **Sample** | **Age** | **HPV type** | **TCT** | **Pathological Diagnosis Results** |
| --- | --- | --- | --- | --- | --- |
| Cervicitis | A03 | 31 | HPV18 | DNA aneuploidy | Cervicitis |
|  | A13 | 34 | HPV16 | Negative | Cervicitis |
|  | A17 | 18 | HPV16 | Negative | Cervicitis |
|  | A14 | 47 | HPV56 | LSIL | Cervicitis |
|  | A25 | 27 | HPV18 | Negative | Cervicitis |
| CIN I | A01 | 33 | HPV52 | ASCUS | CINI |
|  | A20 | 42 | HPV58 | Negative | CINI |
|  | A21 | 23 | HPV39 | LSIL | CINI |
|  |  |  | HPV58 |  |  |
|  | A02 | 53 | HPV56 | ASCUS | CINI |
|  | A23 | 28 | HPV56 | LSIL | CINI |
| CIN II | A05 | 42 | HPV52 | HSIL | CINI-IIP16+ |
|  | A07 | 63 | HPV56 | / | CINII |
|  | A12 | 30 | HPV31 | LSIL | CINI-IIP16+ |
|  | A16 | 33 | HPV33 | LSIL | CINII |
|  | A26 | 45 | HPV18 | Negative | CINII |
| CIN III | A10 | 33 | HPV16 | Negative | CINIII |
|  | A15 | 42 | HPV58 | LSIL | CINIII |
|  | A24 | 36 | HPV16 | Negative | CINII-IIIP16+ |
|  |  |  | HPV68 |  |  |
|  | A09 | 43 | HPV59 | LSIL | CINII-IIIP16+ |
|  | A27 | 39 | HPV16 | HSIL | CINIII |

Table S4: Break points in gene element regions

| **Gene element region** | **Observed** | **Expected1** | **Expected2** | **Expected3** | **Expected4** | **Expected5** | **Expected Average** | ***p* value** |
| --- | --- | --- | --- | --- | --- | --- | --- | --- |
| Exonic | 193 | 223 | 214 | 204 | 196 | 181 | 204 | 9.83E-03 |
| Intronic | 6869 | 6613 | 6483 | 6390 | 6486 | 6585 | 6511 | 2.23E-03 |
| Intergenic | 10157 | 10357 | 10476 | 10569 | 10497 | 10371 | 10454 | 3.59E-03 |
| ncRNA | 1071 | 1171 | 1192 | 1163 | 1211 | 1200 | 1187 | 1.35E-04 |
| UTR | 231 | 218 | 217 | 262 | 220 | 251 | 234 | NS |
| Upstream-1kb | 169 | 113 | 123 | 117 | 107 | 107 | 113 | 3.02E-05 |
| Downstream-1kb | 124 | 130 | 124 | 118 | 111 | 120 | 121 | NS |

UTR=untranslated region

Table S5: The top20 GO terms in each group

| **Group** | **GO term** | **ID** | **Description** | **Gene Ratio** | **Bg Ratio** | ***p* value** | **p.adjust** | **q value** | **Gene ID** | **Count** |
| --- | --- | --- | --- | --- | --- | --- | --- | --- | --- | --- |
| Cervicitis | BP | GO:1902041 | regulation of extrinsic apoptotic signaling pathway via death domain receptors | 5/264 | 50/18903 | 0.000648815 | 0.745065 | 0.745065 | 2932/3958/407007/6423/7128 | 5 |
|  | BP | GO:1902042 | negative regulation of extrinsic apoptotic signaling pathway via death domain receptors | 4/264 | 31/18903 | 0.000869568 | 0.745065 | 0.745065 | 2932/407007/6423/7128 | 4 |
|  | BP | GO:0050907 | detection of chemical stimulus involved in sensory perception | 16/264 | 477/18903 | 0.001151274 | 0.745065 | 0.745065 | 1472/144124/128367/4991/81318/81309/119694/390081/390083/282775/219464/390148/8590/128366/26494/5330 | 16 |
|  | BP | GO:0046329 | negative regulation of JNK cascade | 4/264 | 39/18903 | 0.00208297 | 0.745065 | 0.745065 | 2218/6423/153769/140885 | 4 |
|  | BP | GO:0050911 | detection of chemical stimulus involved in sensory perception of smell | 14/264 | 431/18903 | 0.003086208 | 0.745065 | 0.745065 | 144124/128367/4991/81318/81309/119694/390081/390083/282775/219464/390148/8590/128366/26494 | 14 |
|  | BP | GO:0017157 | regulation of exocytosis | 9/264 | 213/18903 | 0.003131043 | 0.745065 | 0.745065 | 136/640/8912/23332/10675/2932/5865/6814/57617 | 9 |
|  | BP | GO:0043409 | negative regulation of MAPK cascade | 8/264 | 181/18903 | 0.004000081 | 0.745065 | 0.745065 | 1850/2218/5470/6423/153769/140885/6609/65268 | 8 |
|  | BP | GO:1903305 | regulation of regulated secretory pathway | 7/264 | 145/18903 | 0.004316452 | 0.745065 | 0.745065 | 136/640/8912/10675/2932/6814/57617 | 7 |
|  | BP | GO:0042659 | regulation of cell fate specification | 3/264 | 25/18903 | 0.004935066 | 0.745065 | 0.745065 | 8324/57459/6423 | 3 |
|  | BP | GO:0007608 | sensory perception of smell | 14/264 | 457/18903 | 0.005157924 | 0.745065 | 0.745065 | 144124/128367/4991/81318/81309/119694/390081/390083/282775/219464/390148/8590/128366/26494 | 14 |
|  | BP | GO:0032873 | negative regulation of stress-activated MAPK cascade | 4/264 | 51/18903 | 0.005554892 | 0.745065 | 0.745065 | 2218/6423/153769/140885 | 4 |
|  | BP | GO:0070303 | negative regulation of stress-activated protein kinase signaling cascade | 4/264 | 51/18903 | 0.005554892 | 0.745065 | 0.745065 | 2218/6423/153769/140885 | 4 |
|  | BP | GO:0008625 | extrinsic apoptotic signaling pathway via death domain receptors | 5/264 | 85/18903 | 0.006772213 | 0.745065 | 0.745065 | 2932/3958/407007/6423/7128 | 5 |
|  | BP | GO:0016042 | lipid catabolic process | 11/264 | 336/18903 | 0.007859467 | 0.745065 | 0.745065 | 51099/51302/339221/5019/5194/5322/5330/5919/6609/412/6822 | 11 |
|  | BP | GO:0002676 | regulation of chronic inflammatory response | 2/264 | 10/18903 | 0.008120929 | 0.745065 | 0.745065 | 136/7128 | 2 |
|  | BP | GO:0006547 | histidine metabolic process | 2/264 | 10/18903 | 0.008120929 | 0.745065 | 0.745065 | 138199/3176 | 2 |
|  | BP | GO:1900246 | positive regulation of RIG-I signaling pathway | 2/264 | 10/18903 | 0.008120929 | 0.745065 | 0.745065 | 26057/23369 | 2 |
|  | BP | GO:2000726 | negative regulation of cardiac muscle cell differentiation | 2/264 | 10/18903 | 0.008120929 | 0.745065 | 0.745065 | 8324/407007 | 2 |
|  | BP | GO:0071674 | mononuclear cell migration | 8/264 | 206/18903 | 0.008547282 | 0.745065 | 0.745065 | 50848/2625/3958/5641/5919/140885/56833/80024 | 8 |
|  | BP | GO:0007566 | embryo implantation | 4/264 | 59/18903 | 0.009278332 | 0.745065 | 0.745065 | 29974/4318/5618/51673 | 4 |
| CIN I | BP | GO:0016266 | O-glycan processing | 11/1354 | 43/18903 | 0.000166041 | 0.505042 | 0.495037 | 2589/117248/2590/2591/26290/2650/283464/23509/6480/10610/27090 | 11 |
|  | BP | GO:0030166 | proteoglycan biosynthetic process | 13/1354 | 62/18903 | 0.000375311 | 0.505042 | 0.495037 | 658/64377/55790/92126/2135/2300/9653/9957/9956/9951/3479/8509/81031 | 13 |
|  | BP | GO:0014068 | positive regulation of phosphatidylinositol 3-kinase signaling | 15/1354 | 80/18903 | 0.000493822 | 0.505042 | 0.495037 | 1950/2260/2322/3059/3479/653361/4914/5155/80310/5364/55615/4920/145264/23411/219699 | 15 |
|  | BP | GO:0060998 | regulation of dendritic spine development | 12/1354 | 56/18903 | 0.000506442 | 0.505042 | 0.495037 | 4076/65981/26052/100132074/116443/3066/1902/5597/5048/5063/8499/5663 | 12 |
|  | BP | GO:0072124 | regulation of glomerular mesangial cell proliferation | 5/1354 | 11/18903 | 0.000599833 | 0.505042 | 0.495037 | 655/1958/5155/80310/7490 | 5 |
|  | BP | GO:0036124 | histone H3-K9 trimethylation | 6/1354 | 17/18903 | 0.000830507 | 0.505042 | 0.495037 | 4204/55011/23411/6839/79723/10782 | 6 |
|  | BP | GO:0051962 | positive regulation of nervous system development | 35/1354 | 279/18903 | 0.00086175 | 0.505042 | 0.495037 | 575/84079/429/673/4076/65981/147381/63950/1813/1959/2049/7976/3066/3280/3553/3688/3976/9175/4204/27020/9423/4914/9253/5048/5063/5080/5364/6009/6498/22865/6664/26011/7161/284403/7516 | 35 |
|  | BP | GO:0018210 | peptidyl-threonine modification | 19/1354 | 120/18903 | 0.000871265 | 0.505042 | 0.495037 | 90/566/655/51719/199731/817/983/57214/11113/8445/1950/285203/2589/2590/2591/28996/5580/29110/7048 | 19 |
|  | BP | GO:0072110 | glomerular mesangial cell proliferation | 5/1354 | 12/18903 | 0.000967785 | 0.505042 | 0.495037 | 655/1958/5155/80310/7490 | 5 |
|  | BP | GO:0050769 | positive regulation of neurogenesis | 30/1354 | 231/18903 | 0.001124463 | 0.505042 | 0.495037 | 84079/429/673/4076/65981/63950/1813/1959/7976/3066/3280/3553/3688/3976/9175/4204/27020/9423/9253/5048/5063/5080/5364/6009/6498/6664/26011/7161/284403/7516 | 30 |
|  | BP | GO:0014065 | phosphatidylinositol 3-kinase signaling | 21/1354 | 145/18903 | 0.001554174 | 0.505042 | 0.495037 | 1950/2260/2322/3059/3479/3667/25948/653361/4914/5155/80310/375033/5291/113791/5364/55615/4920/145264/23411/219699/677 | 21 |
|  | BP | GO:0042063 | gliogenesis | 37/1354 | 310/18903 | 0.001555665 | 0.505042 | 0.495037 | 6833/208/55107/374/429/430/566/6347/983/8941/1021/92737/1959/3066/3280/3553/3976/1902/4204/4323/400746/9423/5048/5076/5080/8929/5309/8613/5534/5663/6009/4920/22800/6664/9580/26011/7161 | 37 |
|  | BP | GO:0045860 | positive regulation of protein kinase activity | 47/1354 | 420/18903 | 0.00156793 | 0.505042 | 0.495037 | 152/154/57679/51378/374/8313/566/658/51719/9607/896/8941/57214/4921/1813/1950/1958/10979/221472/2260/2322/3479/3481/3553/149233/3619/3654/9175/4216/22924/4142/653361/5027/5058/5155/80310/10464/55011/5580/5590/5663/4920/23411/441251/7048/10333/10040 | 47 |
|  | BP | GO:0009101 | glycoprotein biosynthetic process | 38/1354 | 321/18903 | 0.001572922 | 0.505042 | 0.495037 | 26574/79796/658/64377/55790/1603/92126/285203/2134/2135/2300/2589/117248/2590/2591/26290/2650/283464/9653/9957/9956/9951/3479/10905/57134/8509/58505/23509/5663/91869/81031/6646/6480/10610/27090/6489/54790/200424 | 38 |
|  | BP | GO:0006029 | proteoglycan metabolic process | 15/1354 | 90/18903 | 0.001740737 | 0.505042 | 0.495037 | 81792/11173/658/64377/55790/92126/2135/2300/9653/9957/9956/9951/3479/8509/81031 | 15 |
|  | BP | GO:0070997 | neuron death | 42/1354 | 368/18903 | 0.001871486 | 0.505042 | 0.495037 | 208/317/429/598/673/6347/131450/11113/4921/1735/1958/25793/26281/2558/2668/28996/3162/10525/3672/3725/3745/4204/4929/4914/5063/10038/5309/5459/5663/5700/353116/22800/23411/6548/29110/7015/7068/10333/7009/219699/55737/7516 | 42 |
|  | BP | GO:0071902 | positive regulation of protein serine/threonine kinase activity | 29/1354 | 229/18903 | 0.001984424 | 0.505042 | 0.495037 | 154/57679/658/51719/896/8941/57214/1950/10979/221472/2260/2322/3481/3553/3619/3654/9175/4216/22924/5027/5058/5155/80310/55011/5663/4920/23411/441251/10333 | 29 |
|  | BP | GO:0018212 | peptidyl-tyrosine modification | 43/1354 | 382/18903 | 0.002167631 | 0.505042 | 0.495037 | 90/51378/374/199731/23624/4921/8445/1950/2049/2260/2322/3055/3059/3066/3280/28996/3479/3481/3595/116379/149233/3562/3932/3976/9833/4593/653361/4914/79031/5155/80310/10464/8613/5580/5590/5663/4920/6098/57410/10019/6690/55359/8460 | 43 |
|  | BP | GO:0030213 | hyaluronan biosynthetic process | 5/1354 | 14/18903 | 0.002167731 | 0.505042 | 0.495037 | 57214/1950/3553/5155/55512 | 5 |
|  | BP | GO:0060996 | dendritic spine development | 15/1354 | 92/18903 | 0.002178068 | 0.505042 | 0.495037 | 9912/4076/65981/26052/2049/100132074/116443/3066/1902/5597/5048/5063/8499/5663/158866 | 15 |
| CIN II | BP | GO:0009133 | nucleoside diphosphate biosynthetic process | 7/1557 | 13/18903 | 2.79E-05 | 0.15947 | 0.154783 | 203/204/50808/205/26289/1723/1841 | 7 |
|  | BP | GO:0046940 | nucleoside monophosphate phosphorylation | 6/1557 | 11/18903 | 9.94E-05 | 0.205708 | 0.199662 | 203/204/205/26289/158067/1841 | 6 |
|  | BP | GO:0003279 | cardiac septum development | 21/1557 | 107/18903 | 0.000149999 | 0.205708 | 0.199662 | 652/657/659/84516/54583/2535/2627/57493/182/8543/9794/4488/8131/8828/5754/57167/64220/57057/6926/9321/7474 | 21 |
|  | BP | GO:0002327 | immature B cell differentiation | 6/1557 | 12/18903 | 0.000184951 | 0.205708 | 0.199662 | 25/359948/5591/5897/84447/10758 | 6 |
|  | BP | GO:0006643 | membrane lipid metabolic process | 33/1557 | 209/18903 | 0.000219088 | 0.205708 | 0.199662 | 10351/55750/64781/79603/253782/126410/3995/2720/3073/3074/3696/2531/65258/10825/5007/10400/27315/9487/23556/51604/128869/8398/8612/5408/5660/142891/51097/27293/10558/6484/256435/51046/338596 | 33 |
|  | BP | GO:0060443 | mammary gland morphogenesis | 12/1557 | 46/18903 | 0.000258829 | 0.205708 | 0.199662 | 383/652/1435/780/1969/3488/10765/4488/59350/25928/6926/7474 | 12 |
|  | BP | GO:0002064 | epithelial cell development | 33/1557 | 213/18903 | 0.000312899 | 0.205708 | 0.199662 | 231/546/64919/652/654/440193/9076/1365/2034/1969/22862/137964/2828/57493/3551/182/4478/9480/5906/5908/9693/5914/6093/23094/389015/30812/10653/6781/7111/8626/7414/7474/340481 | 33 |
|  | BP | GO:0030641 | regulation of cellular pH | 18/1557 | 90/18903 | 0.000340541 | 0.205708 | 0.199662 | 480/10159/535/766/11261/1182/51463/120892/5594/54734/7879/6521/57282/6522/9497/6550/389015/10479 | 18 |
|  | BP | GO:0060391 | positive regulation of SMAD protein signal transduction | 7/1557 | 18/18903 | 0.000358211 | 0.205708 | 0.199662 | 652/654/657/659/9688/142/11030 | 7 |
|  | BP | GO:0030004 | cellular monovalent inorganic cation homeostasis | 21/1557 | 114/18903 | 0.00037379 | 0.205708 | 0.199662 | 480/10159/535/766/11261/1182/51463/3759/120892/5594/54734/7879/6558/6507/6521/57282/6522/9497/6550/389015/10479 | 21 |
|  | BP | GO:0033044 | regulation of chromosome organization | 37/1557 | 252/18903 | 0.000414599 | 0.205708 | 0.199662 | 55729/80063/546/9274/9184/7203/22948/10574/990/55755/81620/1063/201161/55743/10664/57520/10459/5594/4171/4609/4683/64151/142/55274/9232/55781/116028/8607/81626/84250/6595/6603/6605/10460/6950/84101/57343 | 37 |
|  | BP | GO:0099504 | synaptic vesicle cycle | 30/1557 | 192/18903 | 0.0004959 | 0.205708 | 0.199662 | 320/382/114781/1138/8973/1182/1488/9829/84062/9815/3356/120892/115677/5028/27445/5906/5908/11069/140730/6093/6455/246213/140679/6616/6809/8871/23025/10497/6844/8674 | 30 |
|  | BP | GO:0110151 | positive regulation of biomineralization | 13/1557 | 56/18903 | 0.00050262 | 0.205708 | 0.199662 | 196527/652/654/657/659/10117/129804/2201/8326/5741/6522/338773/7480 | 13 |
|  | BP | GO:0021879 | forebrain neuron differentiation | 11/1557 | 43/18903 | 0.00055666 | 0.205708 | 0.199662 | 567/64919/55079/388585/4808/8828/4967/23440/9693/9723/57282 | 11 |
|  | BP | GO:0099003 | vesicle-mediated transport in synapse | 32/1557 | 212/18903 | 0.000609 | 0.205708 | 0.199662 | 320/382/114781/50632/1138/8973/1182/1488/9829/1814/84062/9815/3356/120892/115677/5028/27445/5906/5908/11069/140730/6093/6455/246213/140679/6616/6809/8871/23025/10497/6844/8674 | 32 |
|  | BP | GO:0000819 | sister chromatid segregation | 35/1557 | 239/18903 | 0.000614138 | 0.205708 | 0.199662 | 546/9274/9184/990/55755/81620/1060/1063/55743/91782/10664/57520/9928/24137/9113/10459/22919/64151/25836/25936/23047/55274/9232/55781/116028/84250/6595/6603/6605/8243/27127/10460/84101/27183/9525 | 35 |
|  | BP | GO:2000779 | regulation of double-strand break repair | 23/1557 | 135/18903 | 0.000643361 | 0.205708 | 0.199662 | 10097/9274/7913/2074/56776/10459/4255/441161/78990/142/55274/10721/5531/5591/116028/6118/8607/84250/6595/6603/6605/8914/22891 | 23 |
|  | BP | GO:0060390 | regulation of SMAD protein signal transduction | 9/1557 | 31/18903 | 0.000648353 | 0.205708 | 0.199662 | 652/654/657/659/8483/9688/142/11030/10140 | 9 |
|  | BP | GO:0003281 | ventricular septum development | 15/1557 | 74/18903 | 0.000893521 | 0.260565 | 0.252907 | 652/657/659/84516/54583/2535/57493/8543/8131/5754/57167/64220/6926/9321/7474 | 15 |
|  | BP | GO:0055067 | monovalent inorganic cation homeostasis | 26/1557 | 164/18903 | 0.000914106 | 0.260565 | 0.252907 | 134/480/10159/535/766/11261/1182/10699/1814/51463/3759/120892/5594/54734/7879/6558/84561/6507/6521/57282/6522/9497/6550/389015/10479/65125 | 26 |
| CIN III | BP | GO:0051648 | vesicle localization | 28/967 | 213/18903 | 4.41E-06 | 0.023112 | 0.022553 | 55435/8943/26286/23400/388552/26258/84446/9024/84984/8452/1778/54874/8517/547/57234/225689/51168/4647/54820/553115/5537/5728/357/6854/51112/22878/8615/8976 | 28 |
|  | BP | GO:0048199 | vesicle targeting, to, from or within Golgi | 9/967 | 34/18903 | 3.80E-05 | 0.069351 | 0.067675 | 55435/26286/84984/8452/225689/553115/5537/51112/22878 | 9 |
|  | BP | GO:0022408 | negative regulation of cell-cell adhesion | 25/967 | 202/18903 | 3.97E-05 | 0.069351 | 0.067675 | 124872/868/6370/941/6387/2737/23560/3146/3557/3717/284194/10288/5607/4771/4851/4855/55824/80380/5728/5771/5777/4092/23075/59341/84524 | 25 |
|  | BP | GO:0051650 | establishment of vesicle localization | 23/967 | 197/18903 | 0.000193087 | 0.151901 | 0.148231 | 55435/8943/26286/23400/388552/26258/84984/8452/1778/54874/8517/547/57234/225689/51168/4647/54820/553115/5537/357/51112/22878/8976 | 23 |
|  | BP | GO:0045907 | positive regulation of vasoconstriction | 8/967 | 33/18903 | 0.000201404 | 0.151901 | 0.148231 | 146/857/10800/2149/2244/6869/6916/54795 | 8 |
|  | BP | GO:0043476 | pigment accumulation | 5/967 | 12/18903 | 0.000203076 | 0.151901 | 0.148231 | 8943/388552/26258/23682/357 | 5 |
|  | BP | GO:0043482 | cellular pigment accumulation | 5/967 | 12/18903 | 0.000203076 | 0.151901 | 0.148231 | 8943/388552/26258/23682/357 | 5 |
|  | BP | GO:0060070 | canonical Wnt signaling pathway | 31/967 | 310/18903 | 0.000293435 | 0.185709 | 0.181221 | 267/22881/25805/8945/857/25776/122011/23500/55612/8321/11211/2737/2776/27130/8629/3836/26524/4041/130574/1482/4851/261734/90268/5728/3516/6259/6422/388336/54795/79809/9736 | 31 |
|  | BP | GO:0055064 | chloride ion homeostasis | 6/967 | 20/18903 | 0.00036818 | 0.185709 | 0.181221 | 356/6559/57468/9990/6916/7369 | 6 |
|  | BP | GO:0055083 | monovalent inorganic anion homeostasis | 6/967 | 20/18903 | 0.00036818 | 0.185709 | 0.181221 | 356/6559/57468/9990/6916/7369 | 6 |
|  | BP | GO:0060828 | regulation of canonical Wnt signaling pathway | 27/967 | 260/18903 | 0.000390145 | 0.185709 | 0.181221 | 267/22881/25805/8945/857/25776/122011/23500/55612/8321/2737/2776/27130/8629/3836/26524/130574/1482/4851/261734/90268/3516/6422/388336/54795/79809/9736 | 27 |
|  | BP | GO:0030111 | regulation of Wnt signaling pathway | 32/967 | 336/18903 | 0.000557454 | 0.217761 | 0.212498 | 267/22881/25805/8945/857/25776/122011/23500/55612/8321/2737/2776/27130/8629/3836/26524/130574/4776/1482/51701/4851/261734/64359/90268/3516/59343/6422/388336/55959/54795/79809/9736 | 32 |
|  | BP | GO:0016055 | Wnt signaling pathway | 40/967 | 456/18903 | 0.000649402 | 0.217761 | 0.212498 | 267/22881/25805/8945/857/25776/1951/122011/8452/23500/55612/8321/11211/2737/2776/27130/8629/3836/26524/4041/130574/2011/4776/1482/51701/4851/261734/64359/90268/5728/3516/6259/59343/6422/388336/6801/55959/54795/79809/9736 | 40 |
|  | BP | GO:0030900 | forebrain development | 35/967 | 383/18903 | 0.000670463 | 0.217761 | 0.212498 | 333/285025/886/50937/6387/9201/2016/200933/84750/151449/2737/10642/3736/3912/9355/64211/57721/84709/54820/79625/4771/4851/5308/5728/3516/146760/6259/54407/9353/6491/10716/9095/79600/9760/79809 | 35 |
|  | BP | GO:0042532 | negative regulation of tyrosine phosphorylation of STAT protein | 5/967 | 15/18903 | 0.000677012 | 0.217761 | 0.212498 | 857/22876/4771/54625/5771 | 5 |
|  | BP | GO:0030279 | negative regulation of ossification | 8/967 | 39/18903 | 0.000679763 | 0.217761 | 0.212498 | 5167/4851/3516/6422/4092/6779/7166/54795 | 8 |
|  | BP | GO:0198738 | cell-cell signaling by wnt | 40/967 | 458/18903 | 0.000707015 | 0.217761 | 0.212498 | 267/22881/25805/8945/857/25776/1951/122011/8452/23500/55612/8321/11211/2737/2776/27130/8629/3836/26524/4041/130574/2011/4776/1482/51701/4851/261734/64359/90268/5728/3516/6259/59343/6422/388336/6801/55959/54795/79809/9736 | 40 |
|  | BP | GO:0006901 | vesicle coating | 8/967 | 40/18903 | 0.000812104 | 0.235899 | 0.230199 | 8943/26286/8452/225689/553115/5537/51112/22878 | 8 |
|  | BP | GO:0006828 | manganese ion transport | 5/967 | 16/18903 | 0.000943503 | 0.235899 | 0.230199 | 57130/23516/64116/7224/57113 | 5 |
|  | BP | GO:0034504 | protein localization to nucleus | 29/967 | 306/18903 | 0.001069232 | 0.235899 | 0.230199 | 23400/54971/785/868/10575/1408/9201/1871/55612/201163/2737/3320/10527/3717/3836/26524/4000/4771/4851/53371/9818/4928/5356/26953/83593/285190/6672/7295/7507 | 29 |
